# Evaluation of Cervical Intraepithelial Neoplasia Occurrence Following the Recorded Onset of Persistent High-Risk Human Papillomavirus Infection: A Retrospective Study on Infection Duration

**DOI:** 10.3389/fonc.2019.00976

**Published:** 2019-10-01

**Authors:** Cordelle Lazare, Songshu Xiao, Yifan Meng, Chen Wang, Wending Li, Yi Wang, Gang Chen, Juncheng Wei, Junbo Hu, Min Xue, Peng Wu

**Affiliations:** ^1^Cancer Biology Research Center (Key Laboratory of the Ministry of Education), Tongji Medical College, Tongji Hospital, Huazhong University of Science and Technology, Wuhan, China; ^2^Department of Obstetrics & Gynecology, The Third Xiangya Hospital of Central South University, Changsha, China; ^3^School of Public Health, Tongji Medical College, Huazhong University of Science and Technology, Wuhan, China; ^4^Department of Gynecologic Oncology, Tongji Hospital, Tongji Medical College, Huazhong University of Science and Technology, Wuhan, China

**Keywords:** cervical cancer, carcinoma, genital neoplasia, female, gynecology, HPV, CIN

## Abstract

**Objectives:** Persistent high-risk human papillomavirus infection is a major factor in the development of cervical intraepithelial neoplasia and cervical cancer. However, the exact point during this infection that cervical intraepithelial neoplasia develops has eluded researchers. Therefore, we designed a study investigating infection duration between the recorded onset of persistent high-risk human papillomavirus infection and cervical intraepithelial neoplasia development.

**Methods:** Basic descriptive statistics, including the Chi-square test and the Kaplan-Meier method, were used to retrospectively analyze data of 277 women who underwent human papillomavirus genotyping, exhibited persistent high-risk human papillomavirus infection, were cervical cytology negative at enrollment, and developed cervical intraepithelial neoplasia at some point during follow-up.

**Results:** Mean number of cervical cytology and human papillomavirus tests was 2.31 per patient (range: 2–8). Human papillomavirus 16, 52, 58, and 33 accounted for 21.64% (132/610), 21.64% (132/610), 15.90% (97/610), and 10.66% (65/610) of infections, respectively. 42.24% (117/277) and 57.76% (160/277) of women were diagnosed with cervical intraepithelial neoplasia 1 and cervical intraepithelial neoplasia 2+ after persistent high-risk human papillomavirus infection, with mean follow-up times of 18.15 (11.81) and 19.82 (13.31) months, respectively. Cervical intraepithelial neoplasia occurred between 4 and 70 months following the recorded onset of persistent high-risk human papillomavirus infection and 73.65% (204/277) of women developed cervical intraepithelial neoplasia within 24 months.

**Conclusion:** Human papillomavirus 16, 52, 58, and 33 were the most prevalent high-risk human papillomavirus types in a group of women in which the majority developed cervical intraepithelial neoplasia within 24 months of persistent infection.

## Introduction

Cervical cancer (CC) is ranked fourth among leading malignancies in the areas of morbidity and mortality among women worldwide, and persistent infection of any of the 15 high-risk human papillomavirus (HR-HPV) genotypes is necessary for the development of CC and its precursor cervical intraepithelial neoplasia (CIN) (1–3). Persistent HR-HPV infection is seen as the foundation of cervical carcinogenesis. Guidelines have been implemented for the management of women who are HR-HPV persistently positive but cervical cytology negative (4, 5). However, the exact point in time during persistent HR-HPV infection that CIN occurs has eluded researchers.

Several cohort studies have analyzed persistent infection among HR-HPV positive but cervical cytology negative women (6–8). The shared blemish among these studies is that they only focused on human papillomavirus (HPV) infection status at baseline and endpoint, and do not account for the HPV status between these points, thus reducing their conclusions' robustness. Additionally, a recent study by Elfgren et al. concluded that such women will either experience infection clearance or develop cervical intraepithelial neoplasia 2+ (CIN2+) or worse within 6 years (9). However, only 20.5% of enrolled subjects were confirmed to have maintained HR-HPV positivity throughout the study's duration. Such a small percentage of subjects with evidence of maintaining HR-HPV positivity may be insufficient for a convincing conclusion.

With these noted weakness in this area of research, we designed a retrospective study with the aim of determining the interval between the recorded onset of persistent HR-HPV infection and CIN development among a group of women from central south China. To our knowledge, this is the first study of this nature in which the exact infected HR-HPV genotype of all infected women is known from baseline to endpoint and all women displayed persistent HR-HPV from infection onset to CIN diagnosis, making this study the largest to contain these significant qualities.

## Materials and Methods

### Study Population

**Figure 1:**
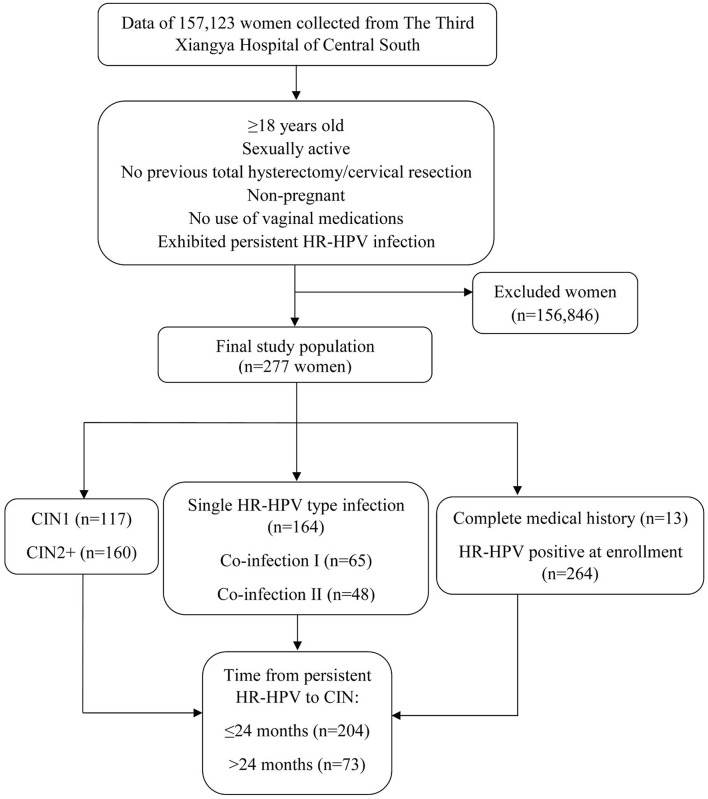
Flowchart representation of subject identification and enrollment.

Clinical data of 157,123 women who underwent HPV genotyping, liquid-based cervical cytology, colposcopy, and biopsy between November 2010 and April 2017 at the Department of Obstetrics and Gynecology of The Third Xiangya Hospital of Central South University, located in the city of Changsha in central south mainland China, were retrospectively analyzed. Inclusion criteria: women ≥18 years; sexually active; no prior history of total hysterectomy or cervical resection; non-pregnant; no use of any vaginal medications at the time of testing (Figure 1). The data of patients analyzed throughout the length of this study was from those who provided informed and written consent to such use during that period. All patients consented to all aforementioned diagnostic testing. Women who were HR-HPV positive but cervical cytology negative were recommended for follow-up testing within 12 months of initial testing. Moreover, women were referred to colposcopy, which included a punch biopsy, after abnormal cervical cytology. From the original subject pool, a total of 277 women who were cervical cytology negative at baseline, exhibited persistent HR-HPV infection throughout follow-up testing and developed CIN were selected and enrolled into this study. Of these, 13 women had complete medical histories showing their progression from being HR-HPV negative, to HR-HPV infected, and finally developing CIN, while the remaining 264 women had no prior medical histories showing an HR-HPV negative state and were HR-HPV positive at enrollment. No further data was collected beyond the point of CIN diagnosis. Women who did not exhibit persistent HR-HPV infection and did not develop CIN were excluded from this study. All excluded subjects were either lost to follow-up, did not exhibit persistent infection, or though exhibited persistent infection there was HPV genotype fluctuation. No cases of CC were included in this study.

### Definitions

Persistent HR-HPV infection was defined as consecutive HR-HPV positive tests for any HR-HPV genotype uninterrupted by negative tests. Single infection refers to the infection with only one HR-HPV genotype, while co-infection refers to the infection of 2 or more HPV genotypes. Co-infection I refers to simultaneous infection of at least HR-HPV types. Co-infection II refers to simultaneous infection of at least 1 HR-HPV and 1 low-risk HPV type.

### Duration of HPV Infection

The date of the first recorded positive HR-HPV test was set as the onset of infection while the date of CIN diagnosis was set as the endpoint for the 264 women who were HR-HPV positive at enrollment. For the remaining 13 women who had complete medical histories infection duration was defined as the time from the date of HR-HPV infection to the date of CIN diagnosis, assuming that the date of infection occurred at the midpoint between consecutive visits with different HPV status (i.e., negative to positive) (10). Thus, appropriate adjustments were made to the infection date for these women. This method could not be applied to the 264 women who were HR-HPV positive at enrollment due to their lack of prior medical histories which recorded an HR-HPV negative state.

### HPV Testing

The 21 human papillomavirus GenoArray Kit (Guangzhou, Guangdong, China) was used for HPV testing. This kit tests for 15 HR-HPV genotypes (16, 18, 31, 33, 35, 39, 45, 51, 52, 53, 56, 58, 59, 66, 68) and 6 low-risk HPV genotypes [6, 11, 42, 43, 44, 81 (CP8304)] (11).

### Cervical Cytology Assessment, Colposcopy, and Biopsy

Cervical cytology reports followed The Bethesda System 2001 (12). The 2001 Bethesda System: Terminology for reporting results of cervical cytology and biopsy samples acquired from colposcopy were analyzed by expert pathologists. All CIN diagnoses were obtained from cervical tissue samples acquired from colposcopy-directed punch biopsy.

### Statistical Analysis

Basic descriptive statistics, including the Chi-square test, and *t*-test were used to summarize data, where data are presented as mean (standard deviation). Cumulative incidence proportions of CIN1, CIN2+, and the 13 women with complete medical histories were evaluated using Kaplan-Meier. Statistical analysis was performed using the SAS software (SAS for Windows, Version 9.4, SAS Institute, USA). All figures showing cumulative incidence proportion were drawn using GraphPad Prism Software (Version 5.0, GraphPad Software, USA).

## Results

Among 157,123 women who underwent the aforementioned testing, 277 women who were cervical cytology negative at baseline, exhibited persistent HR-HPV infection throughout follow-up testing and developed CIN were enrolled in this study. 42.24% (117/277) and 57.76% (160/277) developed CIN1 and CIN2+, respectively. The mean age of women who developed CIN1 and CIN2+ was 41.14 ± 12.04 and 42.82 ± 10.34 years, respectively. The mean number of cervical cytology and HPV tests was 2.31 per woman (range: 2–8). HPV 16, 52, 58, and 33 were the most prevalent HPV genotypes, accounting for 21.64% (132/610), 21.64% (132/610), 15.90% (97/610), and 10.66% (65/610) of infections, respectively, while all other HR-HPV types accounted for 30.16% (184/610). Women diagnosed with CIN1 and CIN2+ had mean follow-up times of 18.15 ± 11.81 and 19.82 ± 13.31 months, respectively.

The estimated interval between the recorded onset of persistent HR-HPV infection and the development of CIN was 4–70 months (Table 1). Categorical analysis was carried out which included single HR-HPV infection, co-infection, and the most prevalent HR-HPV types. Of the 59.21% (164/277) of women who exhibited single HR-HPV, 42.68% (70/164) developed CIN1, and 57.32% (97/164) developed CIN2+ after estimated mean infection times of 17.07 ± 10.03 (range: 4–58) and 19.70 ± 12.17 (range: 6–63) months, respectively. 23.47% (65/277) of women exhibited co-infection I. In this group, 41.54% (27/65) and 58.46% (38/65) of women developed CIN1 and CIN2+ after projected mean infection of 19.22 ± 14.90 (range: 6–63) and 17.92 ± 13.31 (range: 6–56) months, respectively. 17.32% (48/277) of women exhibited co-infection II. 41.67% (20/48) and 58.33% (28/48) of these women developed CIN1 and CIN2+ after probable mean infection of 20.45 ± 13.07 (range: 6–51) and 22.79 ± 16.60 (range: 7–70) months, respectively. When individually analyzed, the mean estimated interval between the recorded onset of persistent HR-HPV infection and the development of CIN among the most prevalent HPV types in this study were quite similar to those of single HR-HPV infection and co-infection.

**Table 1:** Multivariable analysis of the time (month) interval between the recorded onset of persistent HR-HPV infection and CIN development.

	**CIN1**	**CIN2+**	***P*-value**
Single HR-HPV infection			0.14
*N*	70	94	
Mean (SD)	17.07 (10.03)	19.70 (12.17)	
Range (min–max)	4–58	6–63	
Co-infection I			0.71
*N*	27	38	
Mean (SD)	19.22	17.92 (13.31)	
Range (min–max)	6–63	6–56	
Co-infection II			0.60
*N*	20	28	
Mean (SD)	20.45 (13.07)	22.79 (16.60)	
Range (min–max)	6–51	7–70	
HPV 16			0.93
*N*	15	35	
Mean (SD)	16.33 (8.82)	16.11 (8.14)	
Range (min–max)	6–34	7–43	
HPV 52			0.52
*N*	30	19	
Mean (SD)	19.75 (11.13)	22.72 (14.42)	
Range (min–max)	4–58	6–63	
HPV 58			0.62
*N*	9	18	
Mean (SD)	15.33 (8.15)	18.78 (12.87)	
Range (min–max)	6–28	7–48	
HPV33			0.90
*N*	5	17	
Median (IQR)	18 (10–26)	17 (11–35)	
Range (min–max)	7–43	7–46	

*CIN, cervical intraepithelial neoplasia; CIN2+, CIN2/3; HPV, human papillomavirus; HR-HPV, high-risk human papillomavirus; SD, standard deviation; max, maximum; min, minimum; N, number of patients*.

*Co-infection I, simultaneous infection of at least 2 HR-HPV types*.

*Co-infection II, simultaneous infection of 1 HR-HPV type and at least 1 non-oncogenic HPV type*.

**Figure 2:**
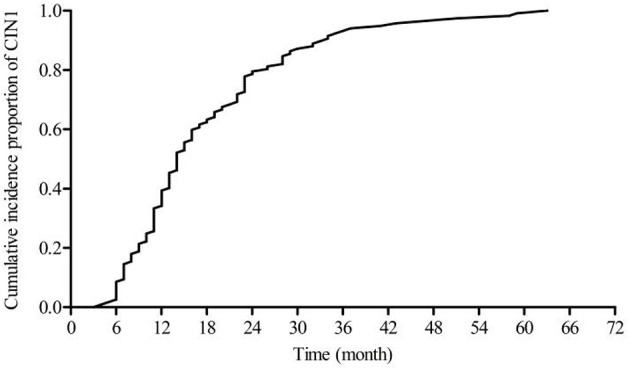
Cumulative proportion of CIN1.

**Figure 3:**
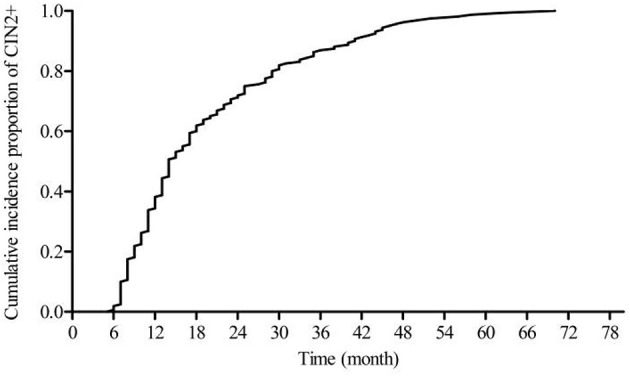
Cumulative proportion of CIN2+.

Figure 2 shows a steadily increasing cumulative incidence proportion of CIN1 until 24 months. Figure 3 shows a similar pattern for CIN2+, as the cumulative incidence proportion steadily increased until 24 months and slows thereafter. Table 2 shows the analysis of the interval between the recorded onset of HR-HPV infection and the development of CIN based on 12-month intervals of <12, 12–23, 24–35, and ≥36 months. 77.77% (91/117) of these women developed CIN1 within an estimated interval of 24 months from the recorded onset of persistent HR-HPV infection, while 22.23% (26/117) were estimated to have developed CIN1 after persistent HR-HPV infection exceeding 24 months. 70.62% (113/160) of CIN2+ women were diagnosed within an estimated 24 months of the recorded onset of persistent HR-HPV infection, while 29.38% (47/113) developed CIN2+ after persistent HR-HPV infection estimated to exceed 24 months. When examined as single infection and co-infection, more than 50% of women in each category were estimated to have developed CIN within 24 months from the noted onset of persistent HR-HPV infection. A total of 73.65% (204/277) of women were proposed to have developed CIN within 24 months following the recorded onset of persistent HR-HPV infection. The influence of age was analyzed among four randomly selected age groups of the cervical cancer screening population. It was observed that in every age group more than 50% of women were estimated to have developed CIN within 24 months of recorded onset of persistent HR-HPV infection, with at least 30% of women in each age group probably developing CIN within 12 months.

**Table 2:** Analysis of the time (month) interval between the recorded onset of persistent HR-HPV infection and CIN development based on 12 month intervals.

	**Time (month)**				***P*-value**
	**<12** ***N* (%)**	**12 ~ 23** ***N* (%)**	**24 ~ 35** ***N* (%)**	**≥36** ***N* (%)**	
CIN					0.36
CIN1	39 (33.33)	52 (44.44)	17 (14.53)	9 (7.70)	
CIN2+	54 (33.75)	59 (36.87)	25 (15.63)	22 (13.75)	
CIN1					0.26
Single HR-HPV infection	25 (35.71)	30 (42.86)	12 (17.14)	3 (4.29)	
Co-infection I	8 (29.63)	14 (51.85)	2 (7.41)	3 (11.11)	
Co-infection II	6 (30.00)	8 (40.00)	3 (15.00)	3 (15.00)	
CIN2+					0.18
Single HR-HPV infection	28 (29.79)	37 (39.36)	18 (19.15)	11 (11.70)	
Co-infection I	19 (50.00)	10 (26.32)	4 (10.53)	5 (13.15)	
Co-infection II	7 (25.00)	12 (42.86)	3 (10.71)	6 (21.43)	
Age (years)					0.43
<30	10 (33.33)	13 (43.33)	3 (10.00)	4 (13.33)	
30–44	52 (33.55)	64 (41.29)	25 (16.13)	14 (9.03)	
45–59	24 (34.78)	29 (42.03)	9 (13.04)	7 (10.14)	
≥60	7 (30.43)	5 (21.74)	5 (21.74)	6 (26.09)	

*CIN, cervical intraepithelial neoplasia; HR-HPV, high-risk human papillomavirus*.

*Co-infection I, simultaneous infection of at least 2 HR-HPV types*.

*Co-infection II, simultaneous infection of 1 HR-HPV type and at least 1 low-risk HPV type*.

**Figure 4:**
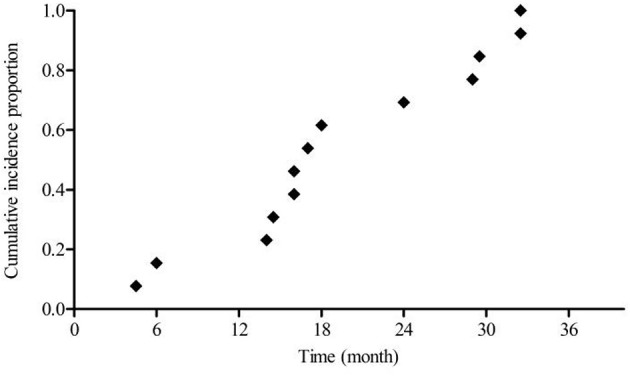
Cumulative proportion of CIN in women with complete medical histories.

Due to the majority of patients in this study being HR-HPV positive at enrollment, it is possible that the overall estimated infection duration leading up to the development of CIN was underestimated. In an effort to compensate for this possible underestimation, the 13 women with complete medical histories among the enrolled 277 cases were individually analyzed. Table 3 provides information on age, number of HPV tests, the interval between tests, type of HPV infection, infection duration, and CIN grade. Mean age was 43.23 ± 11.85 years. 61.54% (8/13) of these women were between the ages of 30 and 44, while 30.77% (4/13) and 7.69% (1/13) of these women, respectively, fell within the 45–59 and >60 years old age groups. The mean interval between HPV tests was 13.3 ± 4.14 months. CIN was observed after mean estimated infection interval of 19.5 ± 9.33 (range: 4.5–32.5) months. Figure 4 shows the cumulative incidence proportion of CIN among these women, after making adjustments based on the aforementioned definition of infection duration applied to this group of women. 69.23% (9/13) were estimated to have developed CIN within 24 months of the recorded onset of persistent HR-HPV infection, while CIN was estimated to have developed after persistent HR-HPV infection exceeding 24 months in only 30.77% (4/13) of women. Hence, infection duration between onset and CIN development among these 13 women is quite similar to that observed among the 264 women who were HR-HPV positive at enrollment.

**Table 3:** Analysis of 13 women with complete medical histories.

**Cases**	**Age at first** **HPV test**	**No. of HPV negative tests**	**No. of HPV positive tests**	**Average interval (months) between tests**	**HPV types(s)**	**Length of infection** **(months)**	**CIN**
1	53	1	3	13	16	29.5	1
2	56	2	3	12	16	29	1
3	33	1	2	25	16/52^a^	6	1
4	42	1	3	7.3	58	16	1
5	31	2	2	15	18	17	1
6	70	1	2	15.5	33	24	1
7	31	2	2	11	16	14	2+
8	31	1	2	15	16/18^a^	32.5	1
9	53	2	2	12	52	18	1
10	45	1	2	13	39/52^a^	14.5	1
11	39	1	2	11	58/53^b^	16	2+
12	38	2	2	10.6	33	32.5	1
13	40	2	1	12.5	18	4.5	1

a *Co-infection I*.

b *Co-infection II*.

*CIN1, cervical intraepithelial neoplasia grade 1; CIN2+, cervical intraepithelial neoplasia grade 2/3; HPV, human papillomavirus*.

## Discussion

The present study evaluated the interval between the recorded onset of persistent HR-HPV infection and the occurrence of CIN, thus reporting several salient findings. First, study subjects aged 19–73 years, representing the general CC screening population with the exception of two women younger than the stipulated age for the commencement of CC screening (13, 14). Second, the mean follow-up time for CIN1 and CIN2+ women was 18.15 ± 11.81 and 19.82 ± 13.31 months, respectively. Third, HPV 16, 52, 58, and 33 were the most prevalent types of high-risk human papillomavirus, similar to the findings of previous studies in this region (15, 16). Fourth, unlike a previous study by Jaisamrarn et al. we observed that co-infection did not individually affect the estimated infection duration from onset to CIN occurrence (17). Fifth, the estimated infection duration from onset to CIN development ranged between 4 and 70 months, with 73.65% of women being diagnosed with CIN within 24 months from baseline.

A number of advances have been made in CC screening over the years. Newer strategies include HPV testing as primary CC screening in women ≥30 years old, owing to the fact that HPV genotyping has better sensitivity and reproducibility compared to cytology, and has increased ability in detecting precancerous lesions (18–20). Recommendations by EUROGIN, 2010 indicate that women ≥30 years old testing positive for HPV be triaged with cervical cytology, followed by colposcopy in the event of abnormal results or follow-up testing in 6–12 months if cervical cytology results are negative, in contrast to follow-up screening in 5 years for HPV negative women (13). Subsequently, the 2015 American Society for Colposcopy and Cervical Pathology (ASCCP) Interim Clinical Guidance recommended that HPV negative women are screened at 3 year intervals, while HPV16/18 positive women are triaged with colposcopy, while those positive for other HR-HPV types are triaged with cervical cytology and subsequently with colposcopy in the event of abnormal cervical cytology results (14, 21). The latter recommendation of immediate colposcopy for HPV16/18 positive women was met with much controversy as the vast majority of HPV infections are transient, possibly prompting unnecessary testing. With that said, this does not diminish the fact that both HPV16/18 have a greater associated risk for CIN/CC than other HR-HPV genotypes, hence the reason for such measures (5, 6, 8, 22, 23). However, both of the aforementioned recommendations lack the ability for high-grade cervical lesion prediction. Due to this, several biomarkers and/or methods are being investigated to determine their suitability in predicting which HR-HPV positive women actually require further management (24). Overall, the CC screening and management of HR-HPV positive women have come a long way, however, there is more to be done.

HPV prevalence has been observed to be higher than the incidence rate of CIN and CC, meaning only a fraction of HR-HPV positive but cervical cytology-negative women develop CIN and CC (25). Due to the transient nature of HPV infections, the majority spontaneously clear within 1–2 years from the onset of infection in the absence of any treatment (26, 27). Nonetheless, irrespective of HPV genotype more than half of the women in this study were estimated to have developed CIN within the same time it takes for most infections to spontaneously clear.

A number of epidemiological studies have been carried out with all drawing the conclusion that HPV prevalence varies based on geography, race, age, difference in surveyed populations, etc. (15, 28). Here, we observed that HPV 16 and 52 had the same prevalence, a finding which is similar with one of our previous studies in this region and another study by Ding et al. conducted in Taiwan (16, 29). Both studies reported that HPV 52 was actually the most prevalent HPV genotype in the evaluated regions, a complete contradiction to previous epidemiological studies (15, 16, 29). These observations consequently make the equal prevalence of HPV 16 and 52 in this analysis plausible. We propose that such findings may be unique to this region, fully supporting the notion that HPV prevalence is a multifaceted dilemma which varies among regions and requires further epidemiological evaluation especially in this region of mainland China.

The associated risk for the progression to CC is directly proportional with the severity of CIN, hence more attention has been paid CIN2+ (30). It is unknown whether progression through the different CIN grades is sequential, whether progression between multiple grades occurs between follow-up thus missing detection of lower grades, or a grade is skipped at some point during the natural history of CC. Irrespective of the type of progression, we observed that both CIN1 and CIN2+ were estimated to occur within the same time frame of 24 months following the recorded onset of persistent HR-HPV infection, emphasizing the need for early detection.

It is worth mentioning that among the 277 women from which data were analyzed there were 30 women <30 years old and 2 of these were <21 years old. Current CC screening guidelines stipulate that CC screening with the use of cervical cytology should commence at 21 years of age, while only women ≥30 undergo co-testing (13, 14). Additionally, our estimated infection duration from onset to CIN development ranged between 4 and 70 months, meaning that patients were observed to have developed CIN at an interval shorter than the interval stipulated by management guidelines for the follow-up testing of HR-HPV positive but cervical cytology-negative women. Such observations highlight mainland China's lack of a standardized CC screening program and fundamentally sheds light on the need for proper regulation (31, 32). Nevertheless, the data analyzed from these women is still valuable as the majority were estimated to have developed CIN within 24 months of the recorded onset of HR-HPV infection.

Limitations of this study include the fact that it was impossible to pinpoint the exact start of persistent HR-HPV infection and time of CIN development. The baseline and endpoint of this study were mere estimations, set as the date of first recorded positive HR-HPV test (except for adjustments made for the 13 patients with complete medical histories) and the date of CIN diagnosis acquired via means of colposcopy punch biopsy, respectively. Additionally, 264 women had medical histories recording HR-HPV positive status, with no prior history of an HR-HPV negative state. Only 13 women had complete medical histories beginning with an HR-HPV negative state. This limitation was minimized upon individual analysis of the 13 women with complete medical histories, which showed that these women developed CIN after an estimated mean infection of 19.5 ± 9.33 (range: 4.5–32.5) months, with 69.23% (9/13) estimated to have developed CIN within 24 months of the documented onset of persistent HR-HPV infection, following the same trend as the other 264 women. This limitation also highlights that HPV infection and the development of CIN occurred before HPV testing and the discovery of CIN by colposcopy punch biopsy, respectively. Interestingly, CIN most probably well before the 24 months noted in most patients presented here. Moreover, it must be noted that the 100% incidence rate of CIN observed in this study is due to all subjects developing CIN at the endpoint. This incidence rate is not representative of the general population, as only a fraction of HR-HPV infected women will actually go on to develop CIN, while even fewer develop CC. Additionally, the retrospective design of this study led to the issue of patients being lost to follow-up. These patients may probably have infection patterns, disease progression patterns, and other characteristics that differ from patients who were not lost to follow-up. Such a limitation may lead to the under or over-estimation of our findings. Moreover, due to this study's retrospective design, we were unable to analyze risk factors for disease progression such as patient lifestyle, marital status, tobacco and alcohol use, immunodeficiency, etc.

In conclusion, we found that HPV 16, 52, 58, and 33 were the most prevalent HR-HPV genotypes among a group of women in which an overwhelming number were estimated to have developed CIN within 24 months from the recorded onset of persistent HR-HPV infection, the same time needed for most HPV infections to spontaneously clear. At this point, it remains unclear whether more frequent testing is required for HR-HPV positive but cervical cytology negative women and whether immediate colposcopy for all women of this group would be beneficial. However, the interesting findings of this study and other epidemiological studies bring the discussion of whether or not CC screening protocols should be standardized as opposed to individualized based on the needs of specific regions to the forefront. We emphasize that there is a strong case for more investigation into current management guidelines for HR-HPV positive but cervical cytology negative women as these guidelines may not provide coverage for all women of this group.

## Data Availability Statement

The datasets generated for this study are available on request to the corresponding author.

## Ethics Statement

The studies involving human participants were reviewed and approved by Ethics Committee of the Third Xiangya Hospital of Central South University (2018-S003), Ethics Committee of Tongji Hospital, Tongji Medical College, Huazhong University of Science and Technology (TJ-IRB20171001). The patients/participants provided their written informed consent to participate in this study.

## Author Contributions

PW and MX conceived and designed this study. CL and SX drafted the manuscript, reviewed all references, and contributed to statistical analysis. YM and CW were responsible for data collection, while WL and YW focused on statistical analysis. GC, JW, and JH were responsible for quality control. PW made critical revisions to the manuscript. All authors gave their comments on the article and approved the final version before submission.

### Conflict of Interest

The authors declare that the research was conducted in the absence of any commercial or financial relationships that could be construed as a potential conflict of interest.

## Abbreviations

CC: cervical cancer
CIN: cervical intraepithelial neoplasia
CIN1: cervical intraepithelial neoplasia grade 1
CIN2+: cervical intraepithelial neoplasia grade 2+
HPV: human papillomavirus
HR-HPV: high-risk human papillomavirus
SD: standard deviation.
